# The global burden and trends of thyroid cancer attributable to high BMI, and the causal impact of obesity: A study integrating GBD 1990–2021 and bidirectional Mendelian randomization

**DOI:** 10.1097/MD.0000000000049119

**Published:** 2026-06-12

**Authors:** Yingji Ma, Bo Li, Ye Tan, Haijun Lu

**Affiliations:** a Department of Radiation Oncology, The Affiliated Hospital of Qingdao University, Qingdao, Shandong, China.

**Keywords:** body mass index, epidemiological trends, Global Burden of Disease, Mendelian randomization, obesity, thyroid cancer

## Abstract

We assessed the global burden of thyroid cancer caused by high body mass index (BMI) from 1990 to 2021 and evaluated whether obesity is genetically supported as a causal risk factor using bidirectional Mendelian randomization (MR). First, we utilized the Global Burden of Disease (GBD) to estimate the deaths, disability-adjusted life years (DALYs), years lived with disability, and years of life lost (YLLs) attributable to high BMI for thyroid cancer, examining age- and sex-specific patterns from 1990 to 2021. Joinpoint regression analyzed temporal trends, and a decomposition analysis was performed to quantify the contributions of population growth, population aging, and epidemiological changes to the burden change. An autoregressive integrated moving average (ARIMA) model was used to forecast changes in the age-standardized death rate (ASDR). Second, we performed bidirectional MR to test whether genetic predisposition to obesity classes influences thyroid cancer risk and whether reverse causation is supported. The GBD analysis showed that high BMI contributed to 5255 global thyroid cancer deaths and 144,955 DALYs in 2021, with a persistently higher burden in females across all age groups. Death and DALY rates for both sexes increased significantly after age 60, with females exhibiting consistently higher rates across all age groups. While absolute numbers of deaths and DALYs increased substantially from 1990 to 2021, age-standardized rates remained relatively stable, and for all metrics women had higher values than men. Average annual percent changes (AAPCs) were found to be higher among males compared to females by joinpoint analysis. Population growth accounted mainly for increasing deaths and DALYs through decomposition analysis. The ARIMA analysis indicates that the level of ASDR among the male population will continue to rise in the future, and it will accelerate after 2025. In contrast, we did not detect a statistically significant causal effect of obesity on thyroid cancer in this MR analysis (Class 1: odds ratio [OR] = 0.82, *P* = .41; Class 2: OR = 0.96, *P* = .84; Class 3: OR = 0.86, *P* = .39). Key limitations include ancestry constraints of the available GWAS instruments and the use of summary-level data.

## 1. Introduction

Thyroid cancer is a type of malignant tumor of the endocrine system with a relatively high 5-year survival rate (98.5%).^[1]^ What is even more worrying is that the incidence rate has shown a sustained upward trend over the past few years.^[2]^ It is forecasted that cases of thyroid cancer worldwide will rise to 44.1% by 2030,^[3]^ which is a matter of serious public health concern. The increase in the incidence of thyroid cancer is associated with multiple risk factors, including genetic factors, environmental influences, and unhealthy lifestyles.^[4]^ Another important factor contributing to the increase is the widespread application of ultrasound technology, which has made the diagnostic process more accessible and efficient.^[5]^ Given the persistently increasing incidence rate, it has become necessary to establish and improve effective treatment procedures. The current standard treatment methods including chemotherapy, immunotherapy, radiotherapy, surgery and molecular targeted therapy have achieved great success in the treatment of early-stage thyroid cancer.^[6]^ Although progress has been made in diagnosis and treatment strategies, there are still challenges in early prevention and effective treatment for advanced patients, which highlights the necessity of continuing research in this field.

Over the past 10 years, an increasing number of studies have shown that obesity and metabolic disorders are an important risk factor for various cancers, and play a significant role in the occurrence and development of cancer.^[6]^ In 2021, approximately 2.11 billion people were affected by obesity, accounting for nearly half of the global population.^[7]^ Meanwhile, the number of cancer cases caused by high BMI has also significantly increased globally.^[8]^ According to the WHO classification, obesity is categorized into 3 classes based on body mass index (BMI): class 1 (30.0–34.9 kg/m^2^), class 2 (35.0–39.9 kg/m^2^), and class 3 (≥ 40.0 kg/m^2^).^[9]^ Thyroid cancer is a typical example among the risks associated with obesity. The research found that for every 5-unit increase in BMI, the risk of thyroid cancer rose by 25%.^[10]^ The promotion of thyroid cancer by obesity involves multiple biological mechanisms. Insulin resistance may promote tumor progression through activation of the insulin-IGF-1 axis. In addition, excess adipose tissue can increase estrogen production, thereby stimulating thyroid cell proliferation. Obesity-related leptin signaling may further enhance tumor invasiveness via the JAK/STAT pathway. These effects are often accompanied by a chronic inflammatory microenvironment that may act synergistically to facilitate tumor development.^[11]^ However, while there is substantial evidence supporting the correlation between obesity and thyroid malignancies, the underlying mechanisms driving this association remain controversial. At present, there are relatively few studies that systematically analyze the age- and sex-specific gender patterns and trends of thyroid cancer caused by high BMI from 1990 to 2021 through multiple indicators (deaths, disability-adjusted life years [DALYs], years lived with disability [YLDs], and years of life lost [YLLs]),^[12]^ including joinpoint regression analysis, decomposition analysis, and autoregressive integrated moving average [ARIMA] prediction models. Global Burden of Disease (GBD) evaluated 371 diseases in 204 countries by establishing a standardized database, and used a unified indicator system to compare health losses among different regions and populations.^[13]^ To address shortcomings in existing research, this study aims to assess the global burden of thyroid cancer attributable to high BMI from 1990 to 2021, utilizing comprehensive data on deaths, DALYs, YLDs, and YLLs provided by the GBD. Descriptive analyses, decomposition analyses, and joinpoint regression analyses were performed to evaluate global, regional, and national disease burden trends from 1990 to 2021, with projections up to 2035 generated using ARIMA models. However, GBD-based attribution primarily quantifies burden at the population level and does not by itself establish causality at the individual level. In order to further strengthen the causal inference beyond the observational association and explore the related mediating mechanisms, the MR method was applied to this study. MR, utilizing genetic variants as instrumental variables, provides a robust framework for establishing causality, thus enhancing the reliability of findings in this area.^[14]^ Previous MR studies have explored the overall causal relationship between BMI and thyroid cancer, but they did not fully investigate the causal relationship among different degrees of obesity.^[15]^ This work seeks to elucidate varied degrees of obesity and thyroid cancer incidence causality, through the application of bidirectional MR method.

This study had 2 complementary objectives: to utilize GBD 1990–2021 data to quantify the burden of thyroid cancer attributable to high BMI, characterize its temporal, demographic, and geographic patterns, and project future trends; and to apply bidirectional Mendelian randomization to investigate the potential causal relationship between BMI and thyroid cancer incidence. Finally, our results are anticipated to contribute high-quality etiologic evidence and public health interventions focusing on obesity as a modifiable risk factor for prevention.

## 2. Methods

### 2.1. Global burden analysis

#### 2.1.1. GBD data sources and definitions

Organized by the Institute for Health Metrics and Evaluation at the University of Washington in the United States, GBD 2021 provides a comprehensive assessment of the health status of 204 countries and territories for the period 1990 to 2021, using a standardized and comparable methodology.^[13,16]^ It systematically quantifies the burden of 371 diseases and injuries through metrics such as incidence, prevalence, mortality, DALYs, and YLDs, and further incorporates an analysis of 88 modifiable risk factors. This study obtained data from the GBD 2021 database to evaluate the global burden of thyroid cancer attributable to high BMI from 1990 to 2021. The extracted metrics included deaths, DALYs, YLLs, and YLDs to assess the burden attributable to high BMI using the Global Health Data Exchange tool (http://ghdx.healthdata.org/gbd-results-tool, accessed: August 8th 2025). Thyroid cancer was defined as code C73 in the International Classification of Diseases 10th revision.^[17]^ High BMI is defined as a BMI ≥ 25 kg/m^2^ in this study.^[18]^ Both sexes were included, and age stratification was conducted in 5-year intervals ranging from 20 to ≥ 95 years, incorporating both aggregated “all ages” and age-standardized metrics in order to ensure comprehensive coverage for trend analysis.

#### 2.1.2. Descriptive analysis

The burden was assessed from 4 key indicators: mortality, DALYs, YLLs, and YLDs. Both absolute numbers and age-standardized rates (per 100,000 population) were computed for all indicators, with stratification by gender and age to assess the changes and distribution of the burden. The specific steps are as follows: Firstly, the absolute numbers and age-standardized rates of global deaths, DALYs, YLDs, and YLLs are presented through the cross-sectional data of 2021. Secondly, the population distribution characteristics of disease burden are analyzed by stratifying by gender and age group. Finally, the trend changes of the absolute numbers and age-standardized rates of each indicator from 1990 to 2021 are shown.

#### 2.1.3. Joinpoint regression analysis

Joinpoint regression was applied to identify significant changes in trends over time which was first proposed by Kim in 2000.^[19]^ The joinpoint model estimates inflection points where trends change direction or magnitude, allowing for the calculation of annual percent change (APC) and AAPC.^[19]^ The model fits an initial linear trend, followed by permutations of the data to detect potential infection points, after which the AAPC is computed as a weighted average of the APCs from each segment. In the Joinpoint regression analysis, the maximum number of joinpoints was set to 5, depending on the length of the time series, following the standard recommendations of the Joinpoint Regression Program. Model selection was based on the Monte Carlo permutation test, which identifies the optimal number of joinpoints by comparing models with different numbers of joinpoints at a significance level of 0.05.

#### 2.1.4. ARIMA model analysis

This study is based on the ASDR data of GBD from 1990 to 2021. Time series analysis and prediction are conducted using the ARIMA model.^[20]^ The model is established using the Box-Jenkins method. Firstly, the stationarity of the sequence is tested, and if necessary, differencing is performed. The model form is identified using the autocorrelation function and partial autocorrelation function, and the optimal parameter combination (p, d, q) is determined based on the Akaike information criterion.^[21]^ For the male population, an ARIMA (0,1,1) model with drift was selected as the final model, whereas an ARIMA (0,1,0) model was identified for the female population. Model adequacy was evaluated using residual diagnostics, including inspection of residual ACF plots and assessment of residual autocorrelation. No significant residual autocorrelation was observed in either model, supporting the adequacy of the fitted models. A summary of residual diagnostic results for the final ARIMA models is provided in the Supplementary Figure 1, Supplemental Digital Content 1. To assess the stability of the ARIMA forecasts, sensitivity analyses were conducted by fitting models to truncated time series and comparing short-term forecasts with observed values; forecast trends were also visually compared with simple linear trend extrapolations to ensure consistency in the overall direction of change. The mortality trend for the period 2022 to 2035 was predicted based on the final model, and 95% confidence intervals (CIs) were calculated to assess the uncertainty of the prediction, which are displayed as shaded areas in Figure 6.

**Figure 1.**
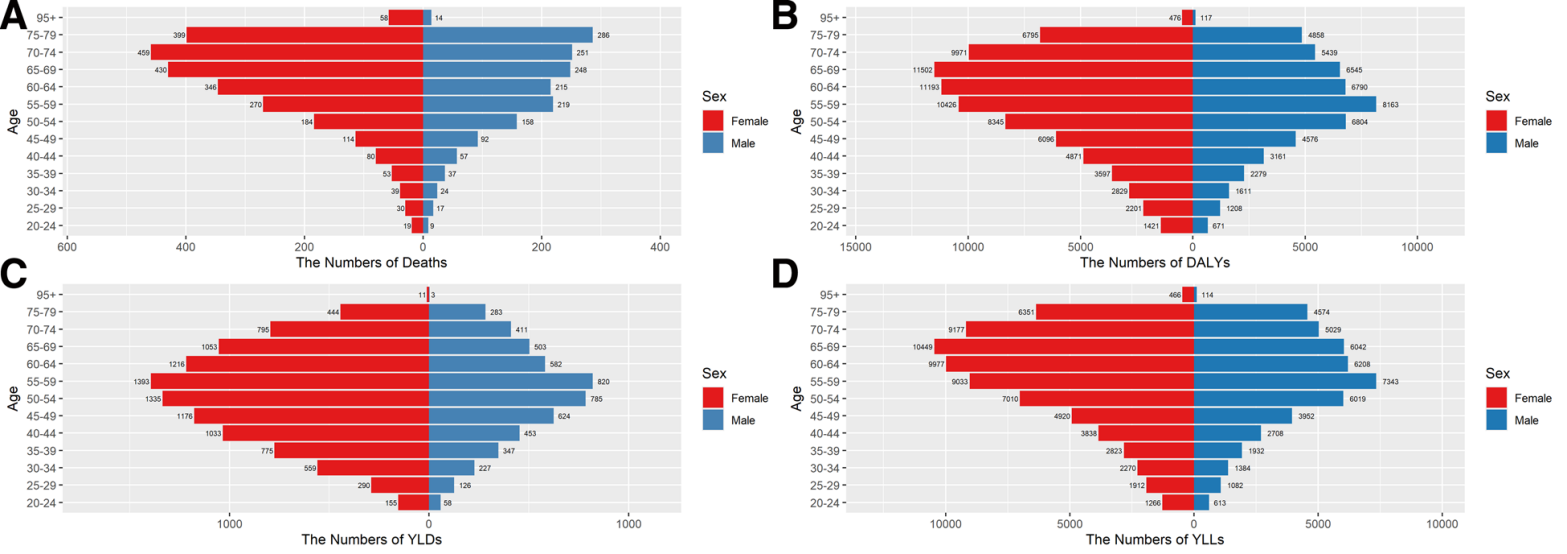
Age and sex distribution of deaths (A), DALYs (B), YLDs (C), and YLLs (D) attributable to high BMI.

**Figure 2.**
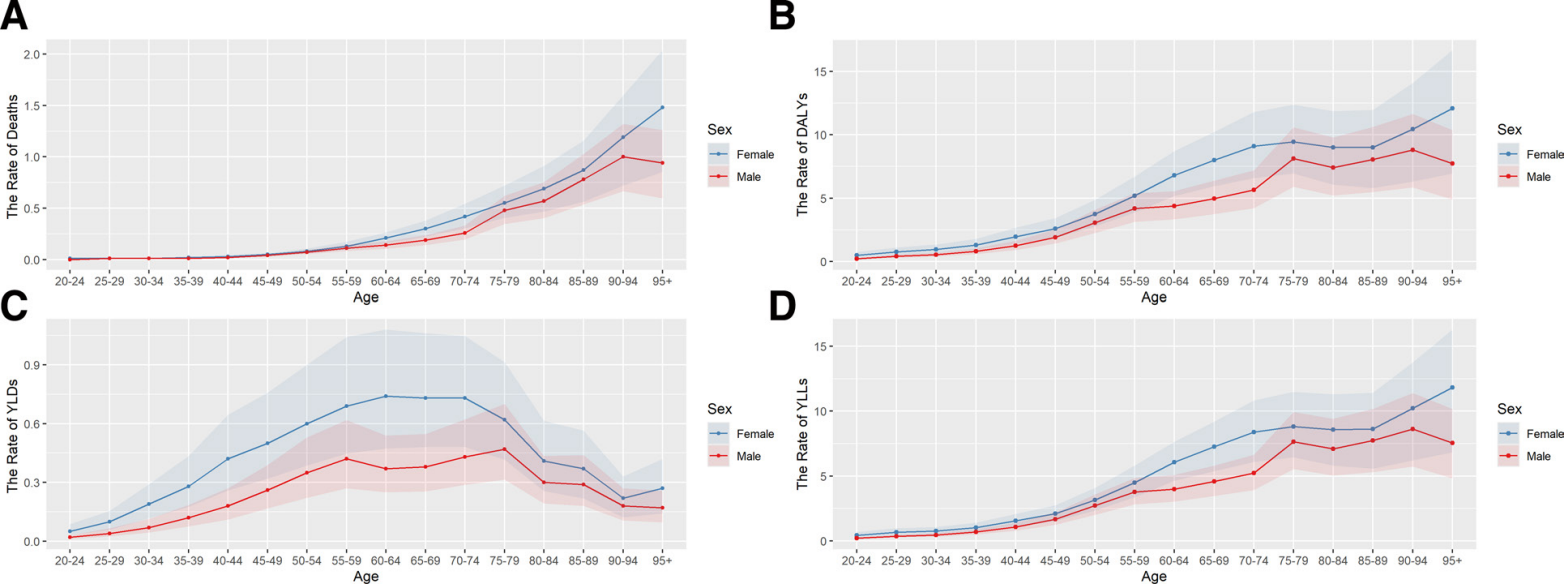
Age-specific rates of thyroid cancer burden attributable to high BMI by sex in Global in 2021, including (A) deaths, (B) DALYs, (C) YLDs, and (D) YLLs.

**Figure 3.**
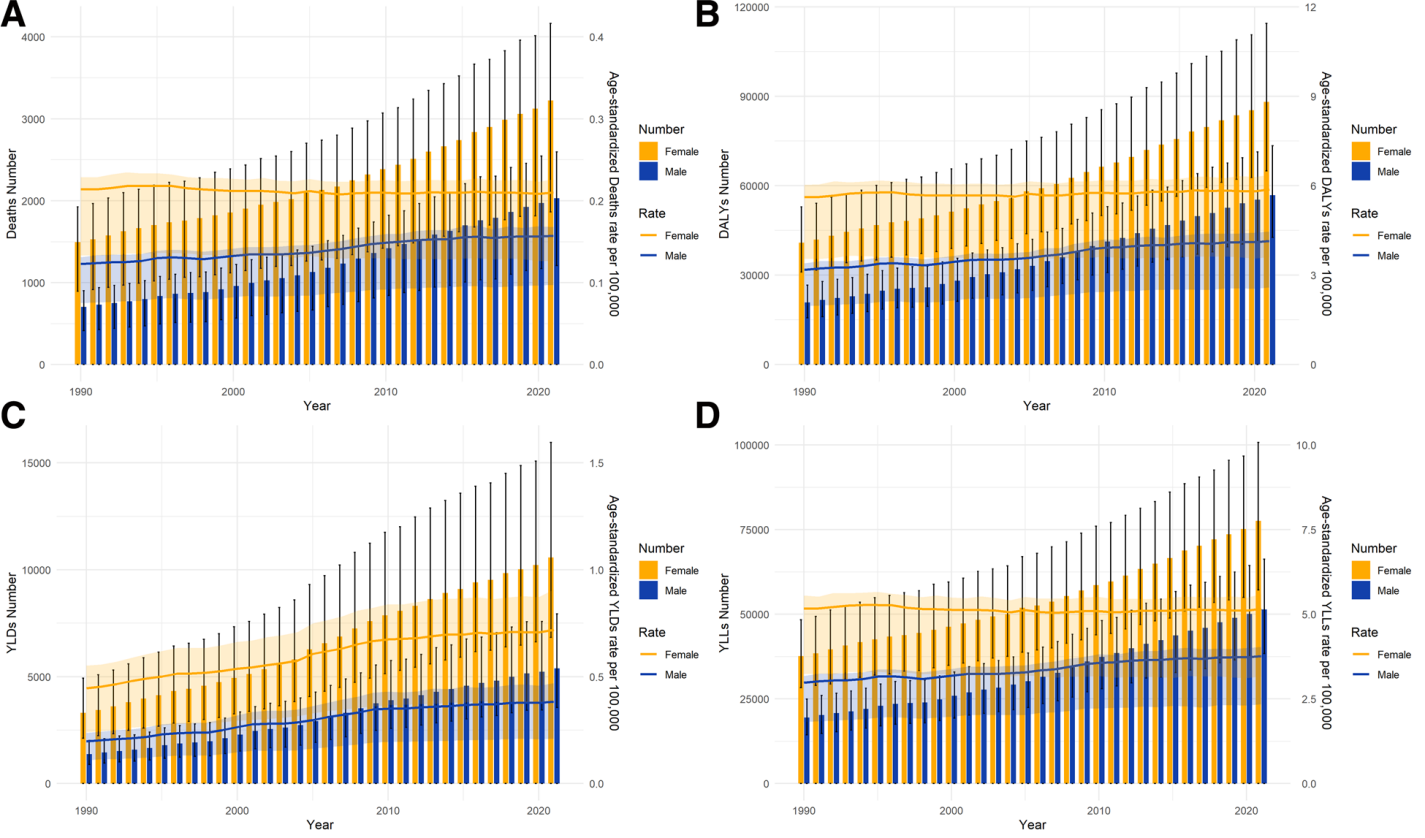
Trends in the number and age-standardized rates of deaths, DALYs, YLDs, and YLLs attributable to high BMI in thyroid cancer in Global, 1990–2021, by sex. (A) The number of deaths and age-standardized death rates. (B) The number of DALYs and age-standardized DALY rates. (C) The number of YLDs and age-standardized YLD rates. (D) The number of YLLs and age-standardized YLL rates.

**Figure 4.**
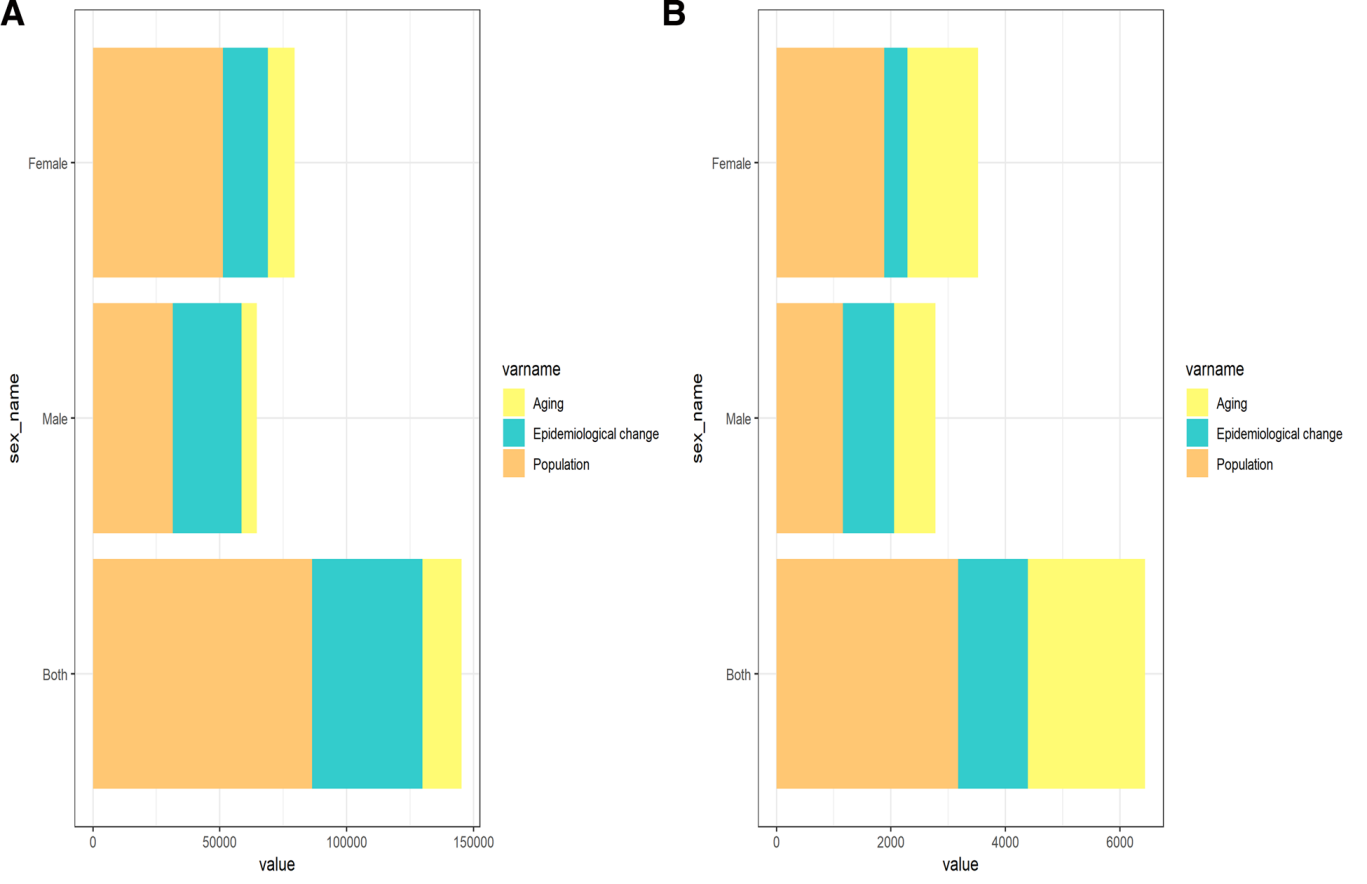
Decomposition analysis of changes in deaths and DALYs attributable to high BMI in thyroid cancer in Global from 1990 to 2021, by aging, epidemiological changes, and population growth. (A) Decomposition of changes in deaths. (B) Decomposition of changes in DALYs.

**Figure 5.**
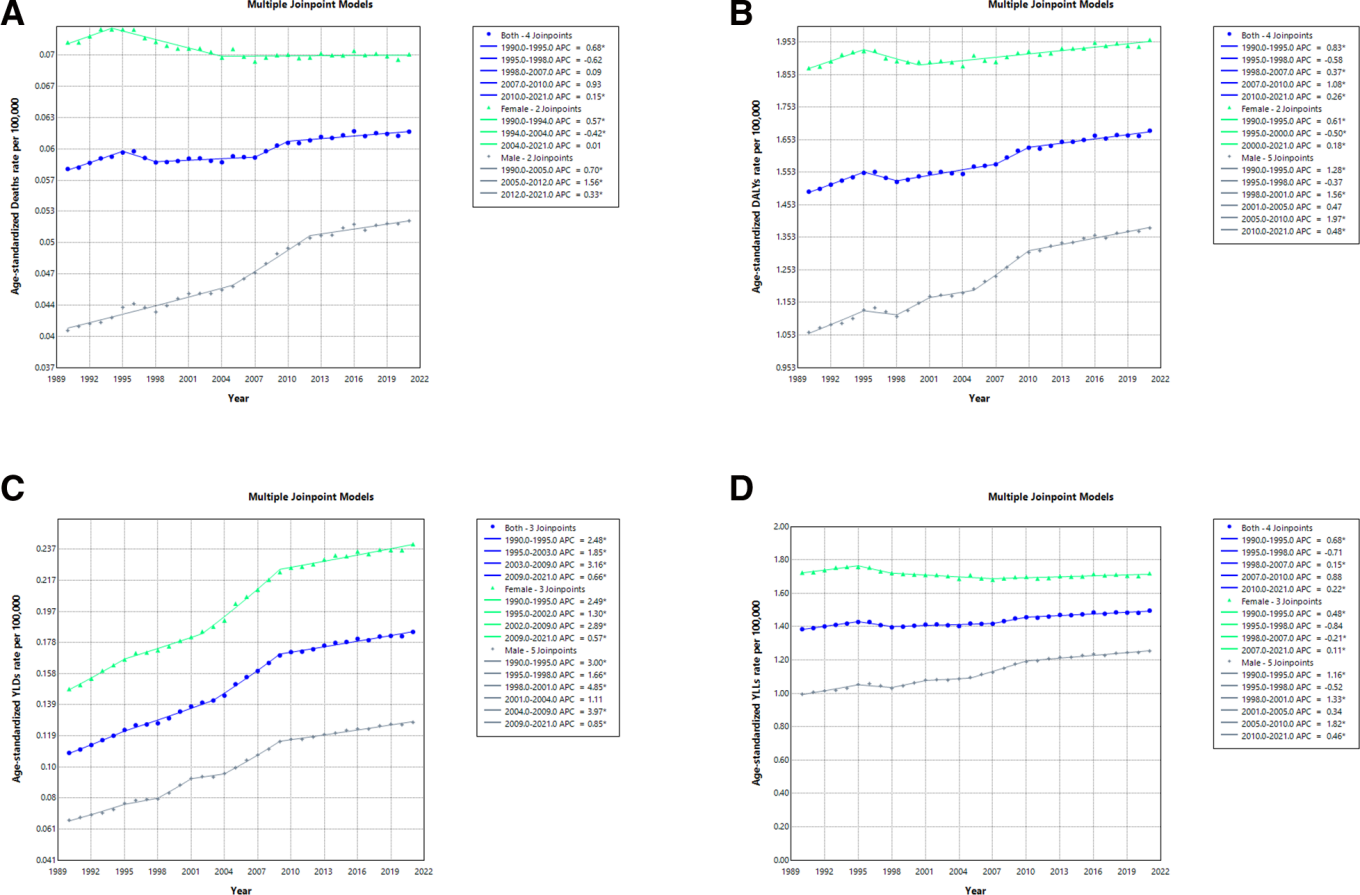
Joinpoint analysis of age-standardized rates for deaths, DALYs, YLDs, and YLLs. (A) The trend in age-standardized death rates. (B) The trend in age-standardized DALYs rate. (C) The trend in age-standardized YLDs rate. (D) The trend in age-standardized YLLs rate.

#### 2.1.5. Decomposition analysis

This study employed the demographic decomposition analysis method to assess the independent contributions of the 3 major drivers to the changes in the global burden of thyroid cancer caused by high BMI from 1990 to 2021. Using standard demographic techniques, the total changes in deaths and DALYs were decomposed into 3 components: the effect of population aging (reflecting changes in the age structure), the effect of population growth (indicating changes in the total population size), and the effect of epidemiological changes (referring to the changes in disease rates after excluding population dynamic factors).^[22]^ This method can accurately distinguish the relative impacts of population structure changes and the actual changes in disease risk on the increase in the burden of thyroid cancer caused by high BMI.^[23]^

### 2.2. MR analysis

#### 2.2.1. MR design and data sources

The instrumental variables used for obesity and thyroid cancer were obtained from the IEU Open Genome-wide Association Study Database, including the genome-wide association study (GWAS) datasets ieu-a-90 (obesity class 1, BMI: 30–34.9 kg/m^2^), ieu-a-91 (obesity class 2, BMI: 35–39.9 kg/m^2^), and ieu-a-92 (obesity class 3, BMI ≥ 40 kg/m^2^).^[9]^ The thyroid cancer GWAS datasets utilized was ieu-a-1082. All analyses strictly followed the 3 core assumptions of the MR analysis: The instrumental variable is strongly correlated with the exposure factor (obesity); the instrumental variable is independent of confounding factors; the instrumental variable affects the outcome only through the exposure factor.

#### 2.2.2. Instrumental variable selection and quality control

To ensure that the instrumental variables meet the basic assumptions of the MR analysis, we carried out a rigorous screening process. Firstly, for each obesity level, we extracted single nucleotide polymorphisms (SNPs) that were significantly associated with obesity from the corresponding datasets. For obesity levels 1 and 2, we used the genome-wide significance threshold (*P* < 5 × 10^−8^); since the number of SNPs that reached this threshold in obesity level 3 was limited, we relaxed the threshold to *P* < 5 × 10^−6^ to ensure sufficient statistical power. Secondly, we conducted a linkage disequilibrium clustering analysis (parameter settings: *r*^2^ < 0.001, distance window = 10,000 kb) to exclude highly correlated SNPs. Subsequently, we removed ambiguous palindromic SNPs to ensure the accuracy of allele direction. To further support the independence assumption, the selected instrumental SNPs were screened using publicly available GWAS databases to assess potential associations with known confounders, including smoking-related traits and socioeconomic indicators. No strong or consistent associations were identified. Finally, we calculated the F-statistic for each SNP to assess the strength of the instrumental variable and retained SNPs with F-statistic > 10 to avoid weak instrumental variable bias. The detailed characteristics of all selected instrumental variables are provided in Supplementary Table S1, Supplemental Digital Content 2. To verify the robustness of the results, we conducted a comprehensive sensitivity analysis, including heterogeneity test (Cochran Q), pleiotropy test (MR-Egger intercept method), and leave-one-out sensitivity analysis. However, due to the limited number of genome-wide significant instrumental variables available for specific obesity classes, particularly after stratifying by sex, we did not perform sex-stratified MR analyses to avoid potential bias from weak instruments. Similarly, additional robust methods requiring a larger number of SNPs were not applied where the instrument count was insufficient to ensure statistical reliability.

### 2.3. Statistical analysis

All statistical analyses in this study were performed using RStudio (version 4.4.2). Joinpoint regression analysis was conducted with joinpoint software (version 5.3.0), and MR analyses were primarily executed using the TwoSampleMR package (version 0.6.8). Five methods were applied for MR analyses. The inverse variance weighted (IVW) method was used as the main reference for the MR analysis results, with the remaining 4 methods used for supplementary analyses. Reverse MR Analysis was employed to investigate the potential causal effect of genetic predisposition to thyroid cancer on obesity traits. A statistical significance threshold of *P* < .05 indicated a causal relationship between exposure and outcome, and an odds ratio (OR) < 1 indicated a negative association.

## 3. Results

### 3.1. GBD analysis

#### 3.1.1. Global disease burden of thyroid cancer attributable to high BMI in 2021

In 2021, thyroid cancer attributable to high BMI resulted in 5255 deaths globally (3225 in females, 2029 in males), with ASDR of 0.07 per 100,000 in females and 0.05 per 100,000 in males (Table 1). The global burden encompassed 144,955 DALYs, with females bearing a disproportionately higher burden (88,120 DALYs) compared to males (56,835 DALYs). ASDR were 1.96 per 100,000 in females versus 1.38 per 100,000 in males. YLDs totaled 15,968 globally, with female-to-male distribution of 10,576 versus 5393, respectively. Age-standardized YLD rates demonstrated a nearly 2-fold difference between sexes (0.24 per 100,000 in females vs 0.13 per 100,000 in males). YLLs contributed 128,986 to the total burden, with females accounting for 77,544 YLLs compared to 51,442 in males. The corresponding age-standardized YLL rates were 1.72 per 100,000 in females and 1.25 per 100,000 in males. Figure 1 illustrates the age-specific distribution of thyroid cancer burden attributable to high BMI stratified by sex. Female mortality predominated across nearly all age groups, peaking at ages 70 to 74 years with over 400 deaths. DALY burden was substantially elevated in females, particularly between ages 55 to 70 years, with peak values exceeding 10,000 DALYs. YLD patterns showed consistent female predominance across the age spectrum, with marked increases after age 40 years. YLL burden was concentrated in females, especially beyond age 40 years, reaching maximum levels in the 65 to 69 age group. These findings demonstrate a pronounced sex disparity in thyroid cancer burden attributable to high BMI, with females experiencing substantially higher rates of mortality, disability, and years of life lost across multiple age strata.

**Table 1:** All-age cases and age-standardized deaths, DALYs, YLDs, and YLLs rates in 2021 for thyroid cancer attributable to high BMI in Global.

Measure	All-ages cases			Age-standardized rates per 100,000 people		
	Total	Male	Female	Total	Male	Female
Deaths	5255 (3914-6653)	2029 (1510-2596)	3225 (2329-4164)	0.06 (0.05–0.08)	0.05 (0.04–0.07)	0.07 (0.05–0.09)
DALYs	144,955 (109,230–184,747)	56,835 (42,680–73,440)	88,120 (64,992–114,469)	1.68 (1.26–2.14)	1.38 (1.04–1.79)	1.96 (1.44–2.55)
YLDs	15,968 (10,370–23,793)	5393 (3556-7943)	10,576 (6834-15,955)	0.18 (0.12–0.28)	0.13 (0.08–0.19)	0.24 (0.15–0.36)
YLLs	128,986 (96,149–162,365)	51,442 (38,389–66,316)	77,544 (57,184–100,732)	1.5 (1.12–1.88)	1.25 (0.94–1.62)	1.72 (1.27–2.24)

DALYs = disability-adjusted life-years, YLDs = years lived with disability, YLLs = years of life lost.

#### 3.1.2. Age and sex differences in disease rates

Figure 2 presents the age-specific burden of thyroid cancer attributable to high BMI stratified by sex. The mortality rates for both males and females began to rise significantly after the age of 60. Females consistently showed higher death rates across all age groups. A similar pattern was observed for DALYs, with rates increasing steadily with age and accelerating beyond age 60, again with females carrying a consistently higher burden than males. For YLDs, females demonstrated higher rates than males throughout the lifespan, particularly in the group aged 60 to 70, highlighting a greater non-fatal disease impact among women. YLL rates increased sharply after age 60, with females experiencing markedly higher years of life lost due to premature mortality compared to males. These findings highlight the disproportionate impact of high BMI-attributable thyroid cancer burden in females, particularly among older adults.

#### 3.1.3. Trends in burden attributable to high BMI in thyroid cancer from 1990 to 2021

As illustrated in Figure 3, the numbers of deaths, DALYs, YLDs, and YLLs attributable to thyroid cancer associated with high BMI all increased markedly from 1990 to 2021. Deaths rose steadily during the study period, with consistently higher values in females, while the ASDR remained relatively stable. DALYs showed a continuous increase in numbers, but the corresponding age-standardized rates fluctuated only slightly, with females carrying a greater burden than males. YLDs displayed a progressive rise in numbers, accompanied by a modest upward trend in age-standardized YLD rates, with a persistent female predominance. The number of YLLs exhibited a pronounced increase, nearly doubling over the 3 decades, and females again contributed more cases than males. Notably, the age-standardized YLL rates remained largely stable throughout this period of substantial numerical growth. These patterns highlight that absolute burden increased substantially, while age-standardized rates remained relatively constant.

#### 3.1.4. Decomposition of changes in deaths and DALYs attributable to high BMI in thyroid cancer

Figure 4 presents the decomposition of changes in deaths and disability-adjusted life years (DALYs) attributable to high BMI in thyroid cancer from 1990 to 2021 at the global level. The total change in deaths and DALYs represents the sum of contributions from population growth, population aging, and epidemiological change. For deaths, population growth was the largest positive contributor to the overall increase, followed by epidemiological change and population aging. The effect of population growth was dominant in both sexes and was slightly more pronounced among females. Population aging also contributed substantially to the increase in deaths, with a greater impact observed in females than in males. Epidemiological change showed sex-specific patterns, with a larger contribution among males compared with females. For DALYs, population growth similarly emerged as the dominant driver of the overall increase, followed by population aging. In contrast, epidemiological change contributed negatively to DALY trends, indicating a reduction in age-specific disease burden over time. However, this favorable epidemiological effect was outweighed by the strong positive contributions of population growth and aging, resulting in a net increase in total DALYs.

#### 3.1.5. Trends in ASRs for thyroid cancer attributable to high BMI in Global, 1990–2021

Joinpoint regression analysis revealed significant temporal variations in age-standardized rates of thyroid cancer burden attributable to high BMI from 1990 to 2021 (Table 2). For both sexes combined, the AAPC showed increasing trends across all measures: mortality rate (AAPC: 0.22, 95% CI: 0.07–0.37), DALYs rate (AAPC: 0.38,95% CI: 0.23–0.53), YLDs rate (AAPC: 1.74, 1.60–1.88), and YLLs rate (AAPC: 0.25, 95% CI: 0.11–0.38). Notably, males demonstrated substantially higher AAPC compared to females in all metrics, with the most pronounced difference observed in YLD rates (male AAPC: 2.18, 95% CI: 1.95–2.42; female AAPC: 1.56, 95% CI: 1.43–1.69). The analysis identified multiple significant joinpoints as shown in Figure 5 indicating changing trends over time. Particularly, males experienced accelerated increases in mortality during 2005 to 2012 (APC: 1.56, 95% CI: 1.18–1.94) and in DALYs rates during 2005 to 2010 (APC: 1.97, 95% CI: 1.61–2.33). Females showed a significant decline in mortality during 1994 to 2004 (APC: −0.42, 95% CI: −0.53 to −0.30) before stabilizing. These findings demonstrate evolving patterns in thyroid cancer burden attributable to high BMI over the past 3 decades, with distinct sex-specific trajectories and significant period variations.

**Table 2:** Trends in age-standardized mortality, DALY, YLD, and YLL rates (per 100,000 persons) among both sexes, males, and females from 1990 to 2021 for thyroid cancer attributable to high BMI in Global.

	Age-standardized mortality rate			Age-standardized DALY rate			Age-standardized YLD rate			Age-standardized YLL rate		
	Period	APC (95% CI)	AAPC (95% CI)	Period	APC (95% CI)	AAPC (95% CI)	Period	APC (95% CI)	AAPC (95% CI)	Period	APC (95% CI)	AAPC (95% CI)
Both	1990–1995	0.68 (0.43–0.93)	0.22 (0.07–0.37)	1990–1995	0.83 (0.58–1.07)	0.38 (0.23–0.53)	1990–1995	2.48 (2.00–2.96)	1.74 (1.60–1.88)	1990–1995	0.68 (0.45–0.90)	0.25 (0.11–0.38)
	1995–1998	−0.62 (−1.69–0.46)		1995–1998	−0.58 (−1.62–0.48)		1995–2003	1.85 (1.57–2.14)		1995–1998	−0.71 (−1.68–0.27)	
	1998–2007	0.09 (−0.03–0.21)		1998–2007	0.37 (0.25–0.48)		2003–2009	3.16 (2.68–3.65)		1998–2007	0.15 (0.04–0.26)	
	2007–2010	0.93 (−0.16–2.03)		2007–2010	1.08 (0.01–2.16)		2009–2021	0.66 (0.54–0.79)		2007–2010	0.88 (−0.09–1.86)	
	2010–2021	0.15 (0.08–0.23)		2010–2021	0.26 (0.19–0.34)					2010–2021	0.22 (0.16–0.29)	
Female	1990–1994	0.57 (0.15–1.00)	−0.05 (−0.12–0.01)	1990–1995	0.61 (0.34–0.88)	0.14 (0.07–0.21)	1990–1995	2.49 (2.04–2.93)	1.56 (1.43–1.69)	1990–1995	0.48 (0.24–0.73)	−0.02 (−0.13–0.10)
	1994–2004	−0.42 (−0.53-−0.30)		1995–2000	−0.50 (−0.87-−0.12)		1995–2002	1.30 (0.98–1.64)		1995–1998	−0.84 (−1.90–0.23)	
	2004–2021	0.01 (−0.04–0.06)		2000–2021	0.18 (0.15–0.21)		2002–2009	2.89 (2.55–3.23)		1998–2007	−0.21 (−0.33-−0.09)	
							2009–2021	0.57 (0.45–0.68)		2007–2021	0.11 (0.06–0.16)	
Male	1990–2005	0.70 (0.60–0.80)	0.79 (0.68–0.90)	1990–1995	1.28 (1.02–1.54)	0.87 (0.70–1.04)	1990–1995	3.00 (2.69–3.32)	2.18 (1.95–2.42)	1990–1995	1.16 (0.91–1.41)	0.76 (0.59–0.93)
	2005–2012	1.56 (1.18–1.94)		1995–1998	−0.37 (−1.47–0.75)		1995–1998	1.66 (0.28–3.06)		1995–1998	−0.52 (−1.61–0.58)	
	2012–2021	0.33 (0.13–0.54)		1998–2001	1.56 (0.44–2.70)		1998–2001	4.85 (3.41–6.31)		1998–2001	1.33 (0.23–2.44)	
				2001–2005	0.47 (−0.08–1.03)		2001–2004	1.11 (−0.31–2.56)		2001–2005	0.34 (−0.21–0.89)	
				2005–2010	1.97 (1.61–2.33)		2004–2009	3.97 (3.50–4.44)		2005–2010	1.82 (1.48–2.17)	
				2010–2021	0.48 (0.41–0.56)		2009–2021	0.85 (0.77–0.94)		2010–2021	0.46 (0.38–0.53)	

BMI = body mass index, DALYs = disability-adjusted life-years, YLDs = years lived with disability, YLLs = years of life lost.

#### 3.1.6. Trends and projections of ASDR in male and female populations

**Figure 6.**
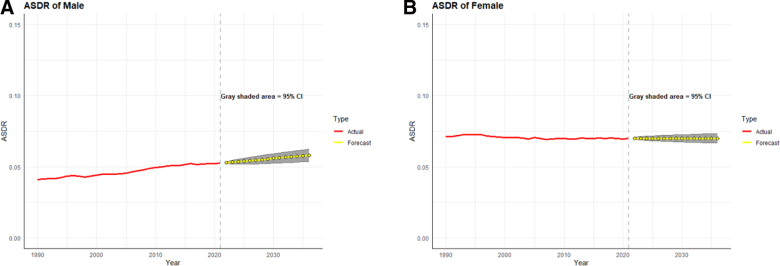
Trends and projections in ASDR for male and female populations from 1990 to 2035. Solid lines represent observed values, while dashed lines indicate projected trends. Gray shaded areas around the projection lines represent the 95% CI. (A) The ASDR for males. (B) The ASDR for females.

Figure 6 illustrates the trends in ASDR for males and females from 1990 to 2021, along with future projections using an ARIMA model. The ASDR for males has been gradually increasing over the years, with a consistent upward trend observed from 1990. The forecast trend indicates that this upward trajectory will continue into the future, with a slight acceleration expected after 2025, as reflected by the dashed yellow line and shaded area. The ASDR for females, which has remained relatively stable over the period with only minor fluctuations. The projected trend for females suggests continued stability in the mortality rate in the coming years. This comparison highlights a clear difference in trends between genders, with male death rates expected to keep rising, while female mortality rates are predicted to remain unchanged.

### 3.2. MR analysis results

**Figure 7.**
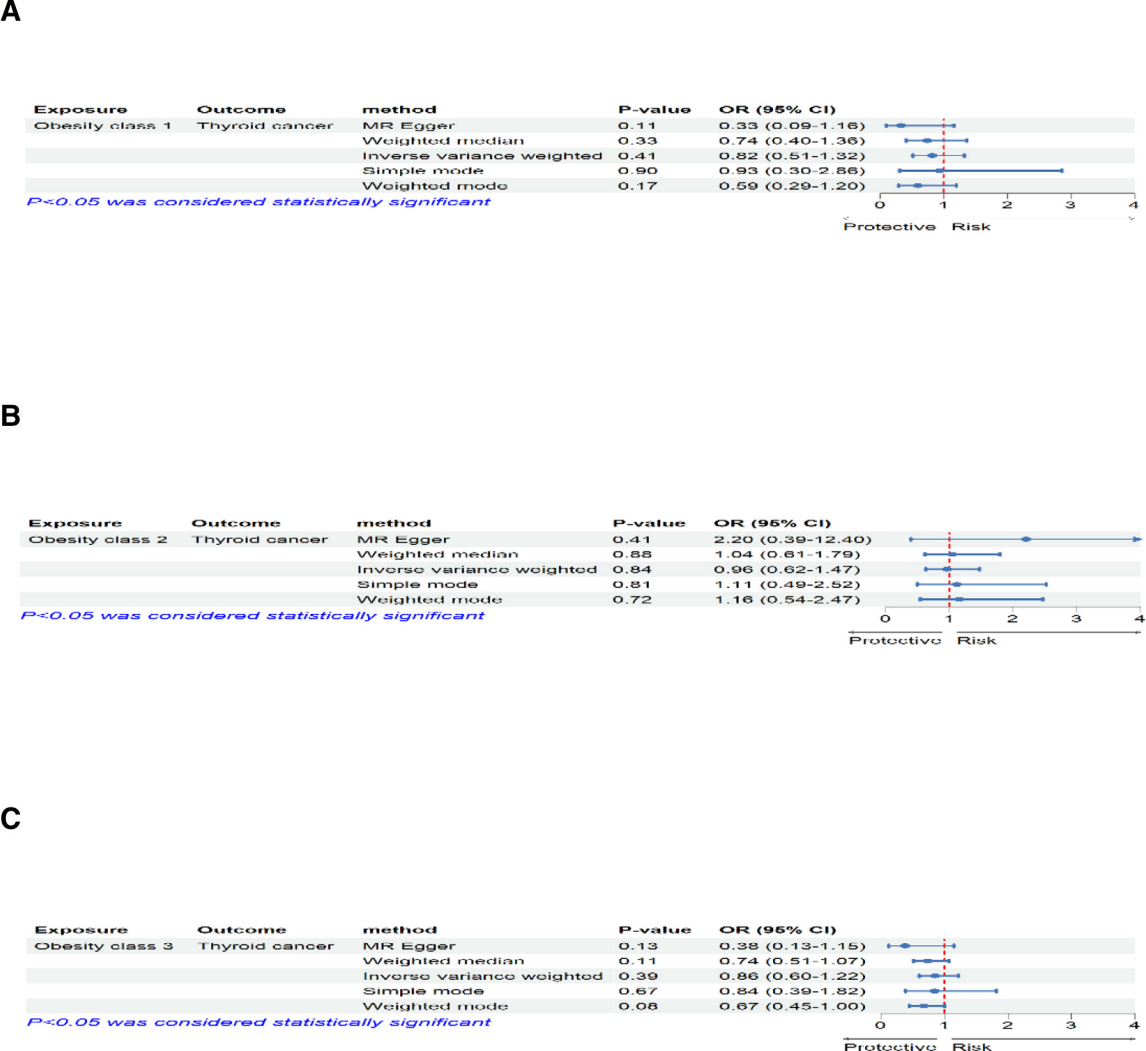
Forest plot of forward Mendelian randomization analysis. (A) Forward MR analysis of obesity class 1 on thyroid cancer. (B) Forward MR analysis of obesity class 2 on thyroid cancer. (C) Forward MR analysis of obesity class 3 on thyroid cancer.

As shown in Figure 7, this MR analysis assessed the bidirectional causal relationships between different obesity classes and thyroid cancer. In the primary direction, IVW estimates did not detect statistically significant causal effects of any obesity class on thyroid cancer risk: obesity class 1 (OR = 0.82, 95% CI: 0.51–1.32, *P* = .41), obesity class 2 (OR = 0.96, 95% CI: 0.62–1.47, *P* = .84), and obesity class 3 (OR = 0.86, 95% CI: 0.60–1.22, *P* = .39). The confidence intervals for all estimates included the null value, indicating uncertainty in the magnitude of the causal effects. Sensitivity analyses and pleiotropy tests yielded consistent results, supporting the robustness of these null findings (Supplementary Figures 2 and 3, Supplemental Digital Content 3). In the reverse MR analysis (Supplementary Figure 4, Supplemental Digital Content 4), evaluating the potential effect of thyroid cancer on obesity, IVW estimates similarly did not detect statistically significant causal effects for obesity class 1 (OR = 1.00, *P* = .69) or obesity class 2 (OR = 1.00, *P* = .21). For obesity class 3, although the IVW estimate remained nonsignificant (OR = 1.00, *P* = .37), the MR-Egger method suggested a weak association (*P* = .04); however, this finding should be interpreted with caution, as it may reflect residual pleiotropy rather than a true causal effect. Overall, this bidirectional MR analysis did not identify consistent evidence for statistically significant causal effects between obesity and thyroid cancer in either direction. Nevertheless, given the width of the confidence intervals, modest causal effects cannot be entirely excluded, particularly in the context of limited statistical power to detect small effect sizes.

## 4. Discussion

Our study systematically evaluated the global burden, temporal trends, and future projections of thyroid cancer caused by BMI during the period from 1990 to 2021. Based on the observational epidemiological analysis, we employed a MR approach to further investigate the potential causal relationship between obesity and thyroid cancer. This method aims to investigate whether obesity has a direct causal effect on the risk of developing thyroid cancer. The results indicated that the numbers of deaths and DALYs rose substantially, primarily due to demographic expansion, while age-standardized indicators remained largely unchanged. Women generally bear a more severe burden, and the situation is expected to stabilize in the future. Nevertheless, men indicated upward rising trends and are forecast to show further increases in mortality. MR analyses did not detect a statistically significant causal association between obesity and thyroid cancer risk at the genetic level, and no reverse causal effect of thyroid cancer on obesity was also observed. Our MR analyses did not provide evidence supporting a direct causal effect of obesity on thyroid cancer risk. The discrepancy between the strong observational associations observed in the GBD analysis and the null findings from MR warrants careful interpretation. While residual confounding in conventional observational studies remains a plausible explanation, several alternative factors should also be considered.

The present study demonstrated a significant rise in the absolute number of thyroid cancer attributable to BMI from 1990 to 2021, and the burden of the disease has been steadily rising. In 2021, more than 5000 deaths and nearly 145,000 DALYs were attributed to high BMI-related thyroid cancer globally. The increasing rate of the disease burden of thyroid cancer caused by high BMI was markedly more important than that of all thyroid cancer, highlighting the urgent need to adopt more effective disease management and prevention measures. The increase in disease burden may be closely related to the rise in global obesity rates,^[24,25]^ or it may be associated with the improvement in diagnostic levels and the enhanced awareness of screening.^[26,27]^ A high BMI is a recognized risk factor for several types of cancer, including thyroid cancer.^[15,28]^ Multiple studies have shown a strong correlation between the increase in obesity rates and the rise in cancer incidence.^[29–31]^ One positive aspect is that previous studies have shown that obesity is not associated with more severe pathological features of thyroid cancer.^[32]^ However, despite this increase in absolute numbers, the mortality rate and DALY per 100,000 populations have remained stable when adjusted for age and time. This phenomenon indicates that the sharp increase in the absolute burden is mainly attributed to the structural factors of global population growth and aging. This pattern is consistent with the observations of several other types of cancer.^[33,34]^ In these cancers, population growth is the main driving factor for the increase in case numbers rather than changes in risk characteristics.^[35]^ Furthermore, our research has revealed that the age-standardized rates for mortality and disability were consistently higher in women than in men, and the sex gap was most pronounced in non-fatal outcomes such as YLDs. The age distribution further revealed that both mortality and disability sharply increased after age 60, peaking in women aged 65 to 74. Within this expanding burden, the consistently disproportionate impact on females is a critical finding. Previous studies have found that most thyroid disorders, such as hypothyroidism, nodular goiter and thyroid cancer, have the highest incidence rates among postmenopausal and elderly women.^[36]^ This phenomenon may be caused by the interaction of various factors such as biological factors, access to medical resources, and diagnostic methods. In terms of mechanism, several reasons may explain the observed gender differences. Firstly, obesity increases estrogen production,^[37]^ which promotes the proliferation of thyroid cells and subsequently leads to the generation of cancer cells.^[38,39]^ Additionally, social and behavioral differences, such as health awareness and access to screening, could also partially explain the female predominance in disease burden.^[40]^ The disease burden was greatest among the middle-aged and older population. A multitude of factors contributed to this phenomenon. With advancing age, the body exhibited diminished efficiency in lipid use, resulting in heightened buildup of surplus adipose tissue. Excessive lipid buildup in the body could result in hyperlipidemia and visceral fat accumulation, both associated with numerous cancers.^[41]^ The diagnosis and treatment methods for elderly patients are the same as those for the general population, but the surgical risks and prognosis are worse for elderly patients compared to younger ones.^[42]^ These findings highlight the importance of designing age- and sex-specific strategies for prevention and long-term management, with a particular focus on older women.

Our joinpoint regression analysis revealed that from 1990 to 2021, the AAPCs of thyroid cancer deaths and DALYs were consistently higher in males compared with females. Importantly, mortality in males accelerated between 2005 and 2012, contrasting with a relative stabilization or decline in females during the same interval. This divergence has crucial implications. While females currently bear a greater absolute burden, the relative rate of increase in males may foreshadow a narrowing sex gap in future decades. Such patterns could reflect lifestyle changes in male populations, such as rising obesity prevalence, differential smoking habits, or occupational exposures-that amplify thyroid cancer risk or outcomes.^[43,44]^ Previous studies have found that males with a high BMI often have comorbidities such as cardiovascular diseases, which increases the risk of death.^[45]^ It also suggests that public health messaging and interventions should not exclusively target females but address male populations as an emerging at-risk group. Our ARIMA projections indicate that ASDR in males will continue rising beyond 2025, with evidence of acceleration. In contrast, female mortality rates are expected to remain relatively stable. This forecast carries several practical implications. First, healthcare infrastructure in male-dominated populations may face increasing strain, particularly in countries with already high obesity prevalence. Second, stable female ASDR suggests that prevention and treatment strategies may have reached a plateau in effectiveness, highlighting the need for novel interventions in female populations as well. While predictive models cannot eliminate uncertainty, they serve as valuable tools in cancer epidemiology by revealing potential future trends and allowing policymakers to make adequate preparation.^[46]^ By combining forecasting with real-time surveillance, health systems can better anticipate shifts in thyroid cancer burden and allocate resources in a timely and evidence-based manner.^[47]^ The decomposition analysis showed that the rise in thyroid cancer deaths and DALYs is driven mainly by population growth. This indicates that demographic transitions explain the expanding burden. Because thyroid cancer risk and mortality rise markedly after age 60, aging societies such as those in East Asia and Europe are likely to face disproportionate challenges, even if incidence stabilizes. Although the factor of population is inevitable, preventing obesity remains the most effective modifiable strategy.

In contrast to the epidemiological findings, MR analysis did not find a significant causal effect between obesity and thyroid cancer. The IVW method showed that the OR values of Class 1–3 obesity and thyroid cancer were close to 1, and there was no statistical significance. Reverse MR analysis also did not show that thyroid cancer significantly affected obesity levels. Despite the marginal effect of MR-Egger in severe obesity (Class 3), its reliability is also limited given the pleiotropic effects. Overall, the present study could not verify the causal relationship between high BMI and thyroid cancer at the genetic level. However, this result does not deny an association with epidemiological investigation of GBD, but rather suggests that the relationship may be driven by mediating mechanisms or confounding factors. First, the MR analyses may have been underpowered to detect modest causal effects, particularly if the true effect sizes are small and the number of available genetic instruments is limited for certain obesity classes. Second, BMI is an imperfect proxy for adiposity and does not capture fat distribution or metabolic heterogeneity, such as visceral versus subcutaneous adipose tissue, which may be more relevant to thyroid carcinogenesis. For example, obesity may indirectly promote tumor development through insulin resistance, chronic inflammation, and IGF-1 pathways, but the genetic instrumental variables used in the MR analysis can only reflect the overall BMI level and it is difficult to cover these specific mechanisms. At the same time, the differences in different pathological subtypes of thyroid cancer may also mask the channel-specific effect. Consequently, the absence of a significant causal signal in MR analyses does not preclude more nuanced or context-dependent relationships between obesity and thyroid cancer risk.

The findings of this study also have several important implications. First, considering the substantial societal burden of obesity, obesity prevention must be prioritized as a key cancer prevention strategy, even in the absence of a proven causal link. Second, gender-specific approaches to screening are needed to address the increasing trend of overburden among women and men. Third, policy makers must consider population factors, especially aging populations, when developing health plans. Some limitations of our study must be acknowledged. Firstly, GBD relies on the data reported by each region for model estimation, which may be limited by the quality of basic data and regional differences. Secondly, the over diagnosis of thyroid cancer in the past decade may lead to the overestimation of the disease burden, especially in high-income and developed countries.^[26]^ Third, the MR Analyses were based primarily on genetic data from populations of European ancestry, with uncertainties regarding their applicability to represent other populations. Fourth, we did not conduct sex-stratified analyses or employ all possible robust sensitivity methods. This decision was driven by the relatively small number of valid instrumental variables identified for the specific obesity phenotypes used in this study, which would have compromised the statistical power and increased the risk of weak instrument bias if further stratified or subjected to methods requiring extensive SNP counts. In addition, the differences between different pathological subtypes of thyroid cancer could not be stratified in this study. Future research should focus on clarifying the mechanistic pathways linking obesity and thyroid cancer, potentially through integrative multi-omics approaches, and utilize larger sample sizes specifically for severe obesity subtypes to enable more granular stratified analyses.

## 5. Conclusions

This study underscores the significant and growing global burden of thyroid cancer attributable to high BMI. While observational associations are strong, MR analyses suggest the relationship may not be causal, instead reflecting complex confounding interactions. Given the wide confidence intervals and limited statistical power, modest causal effects cannot be entirely excluded. Nonetheless, the magnitude of the attributable burden-particularly among older females-highlights the pressing need for weight management strategies, gender-specific interventions, and proactive healthcare planning. In an era of global obesity epidemics and aging populations, thyroid cancer prevention and management must remain a public health priority.

## Acknowledgments

The authors thank the members of the GBD 2021 project for their invaluable contributions to this study.

## Author contributions

**Conceptualization:** Yingji Ma, Haijun Lu.

**Data curation:** Yingji Ma, Bo Li.

**Formal analysis:** Ye Tan.

**Investigation:** Yingji Ma, Bo Li.

**Methodology:** Yingji Ma.

**Project administration:** Yingji Ma, Bo Li.

**Software:** Yingji Ma.

**Supervision:** Haijun Lu.

**Visualization:** Yingji Ma.

**Writing – original draft:** Yingji Ma, Bo Li, Ye Tan.

**Writing – review & editing:** Haijun Lu.



**Figure:**
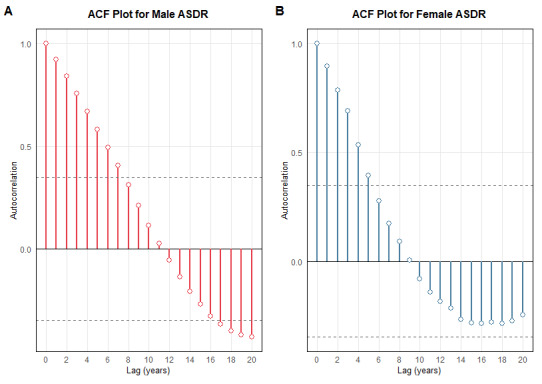


**Figure:**
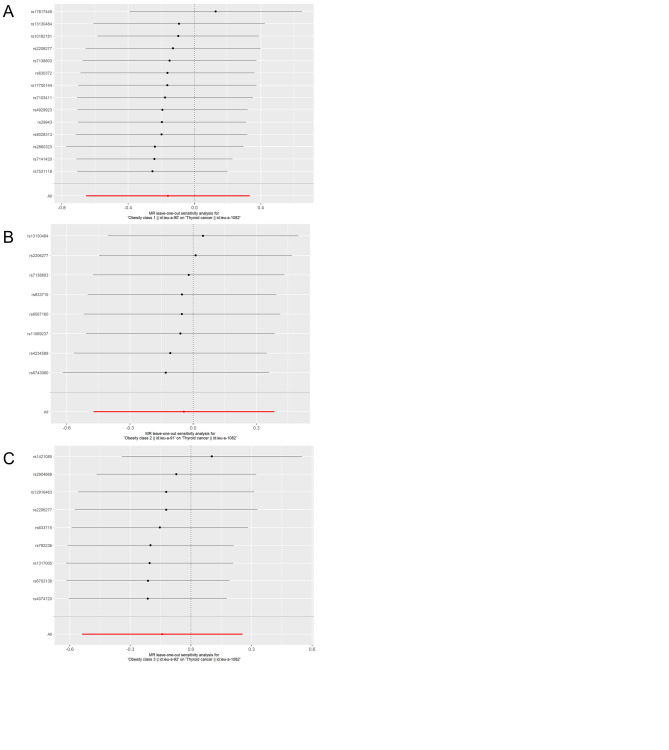


**Figure:**
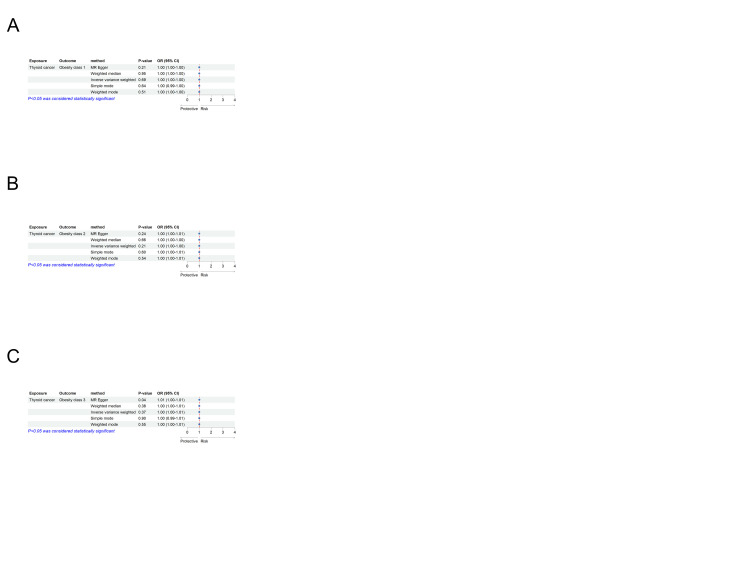


**Figure:**
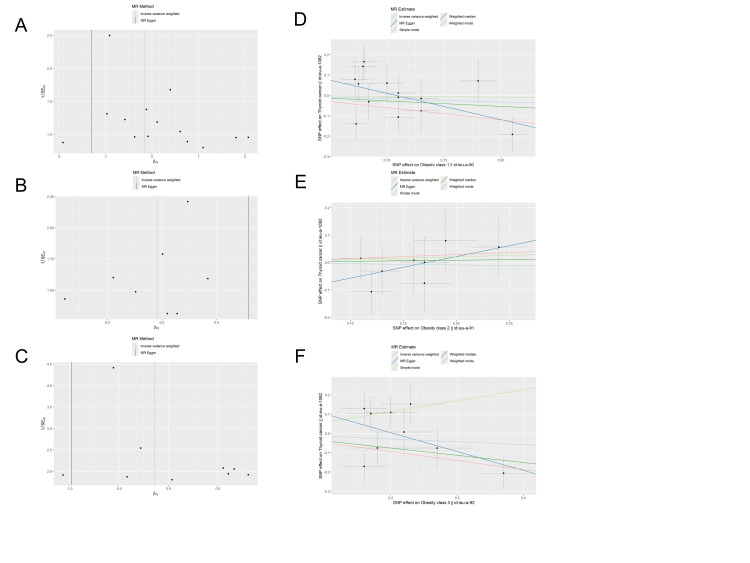

