# Supplementary material for: The global burden and trends of thyroid cancer attributable to high BMI, and the causal impact of obesity: A study integrating GBD 1990–2021 and bidirectional Mendelian randomization
Source: Medicine (Baltimore). 2026 Jun 12;105(24):e49119. doi: 10.1097/MD.0000000000049119 (PMC13268510; doi:10.1097/MD.0000000000049119)
Supplement: Supplementary file 1 [file medi-105-e49119-s002.docx]

| Exposure (Obesity Class) | GWAS ID | P-value Threshold | No. of SNPs (after clumping) | Mean F-statistic | Minimum F-statistic |
| --- | --- | --- | --- | --- | --- |
| Class 1 | ieu-a-90 | 5×10^-8^ | 15 | 69.12 | 29.83 |
| Class 2 | ieu-a-91 | 5×10^-8^ | 11 | 62.53 | 33.52 |
| Class 3 | ieu-a-92 | 5×10^-8^ | 10 | 32.64 | 20.25 |

Supplementary Table1. Characteristics of instrumental variables used in the Mendelian randomization analysis.
